# Venous Air Embolism Leading to Cardiac Arrest in an Infant with Cyanotic Congenital Heart Disease

**DOI:** 10.1155/2012/208430

**Published:** 2012-01-19

**Authors:** Scott C. Watkins, Lewis McCarver, Alicia VanBebber, David P. Bichell

**Affiliations:** Monroe Carell Jr. Children's Hospital at Vanderbilt, Vanderbilt University Medical Center, Nashville, N 37232, USA

## Abstract

Gas emboli, including venous and arterial, are a rare but important complication of pediatric cardiac surgery. They have the potential to have devastating consequences and require prompt recognition and treatment. We present a case of gas embolism occurring in the immediate postoperative period in an infant with cyanotic congenital heart disease after palliative cardiac surgery resulting in cardiopulmonary arrest. The embolism was diagnosed by visualization of air within the vessel creating an airlock and occluding pulmonary blood flow.

## 1. Introduction

Venous and arterial gas emboli are a known and potentially devastating complication of surgical and nonsurgical procedures. Classically associated with neurosurgical procedures performed in the sitting position [1], advances in monitoring and improved awareness of gas embolism have broadened the scope of the problem to include many different surgical and nonsurgical procedures [2]. The number of novel etiologies for gas embolism is large and constantly expanding as technological advances improve recognition and increase the incidence of the problem. Gas emboli in pediatric cardiac surgery have been previously observed during central venous cannulation [3, 4], during sternotomy [5], while opening the heart chambers during cardiopulmonary bypass [6], and due to disconnects in the extracorporeal circuit [6]. We describe an unusual presentation of gas embolism occurring in the immediate postoperative period in an infant with cyanotic congenital heart disease after palliative cardiac surgery resulting in cardiopulmonary arrest.

## 2. Case Description

**Figure 1:**
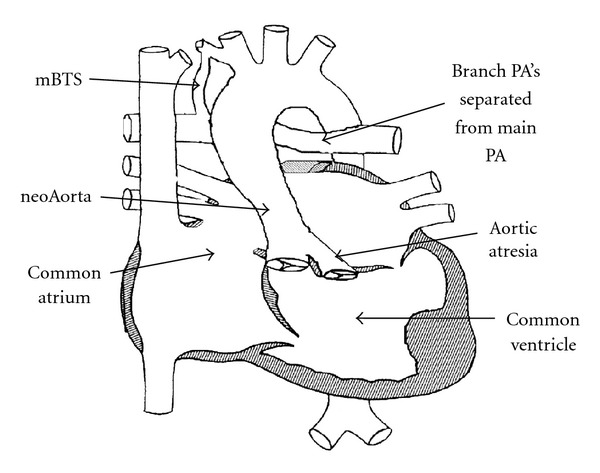
Preoperative anatomy (mBTS: modified Blalock-Taussig Shunt; PA: pulmonary artery).

The patient was a 3-month-old male with cyanotic congenital heart disease consisting of double outlet right ventricle, near absent intraventricular septum, and aortic arch hypoplasia, who had previously undergone a Norwood stage I palliation with 3.5 mm modified Blalock-Taussig shunt (see Figure 1). He presented at 3 months of age for the next stage of palliation, a cavopulmonary anastomosis (bidirectional Glenn). His weight was 5.8 kg (31% percentile) and height was 58 cm (18% percentile). Pulse oximetry demonstrated O_2_ saturations of 70 to 80%, which was appropriate for his lesion. Cardiac catheterization findings included a widely patent arch with estimated normal pulmonary artery (PA) pressures (mean of 12 mmHg in the RPA) by reverse pulmonary vein wedge, a BT shunt widely patent and arising from the innominate artery with flow to the branch pulmonary arteries. The preoperative CXR was unremarkable except for prior sternotomy. A preoperative echocardiogram demonstrated the above described anatomy, insignificant atrioventricular valve insufficiency, a patent modified Blalock-Taussig shunt, and patent proximal branch PA's.

Anesthesia was induced with fentanyl 50 mcg and pancuronium 1 mg, after premedication with intravenous midazolam 1 mg. After endotracheal intubation, anesthesia was maintained with a balanced anesthetic consisting of end tidal isoflurane concentrations of 0.4% and high-dose fentanyl (*∼*100 mcg/kg) in air/oxygen mixture. A left subclavian central venous catheter was placed after induction. A right femoral arterial line was in place from earlier heart catheterization. The patient tolerated anesthetic induction, placement of invasive lines and monitors and the pre-incision period with stable hemodynamics. In addition to standard ASA monitors, bilateral cerebral and somatic oximetry (Somanetics INVOS) was monitored throughout the case. Transesophageal echocardiography (TEE) was not used. It is not our practice to use TEE for cavopulmonary anastomosis procedures or procedures in which the cardiac chambers are not opened.

 Cardiopulmonary bypass (CPB) commenced with bicaval venous cannulation at the inferior vena cava-right atrial (IVC-RA) junction and the innominate vein. The modified Blalock-Taussig shunt was ligated and divided at the initiation of cardiopulmonary bypass. The azygous vein was divided and the superior vena cava was separated from the right atrium. The bidirectional cavopulmonary anastomosis was constructed. The patient was weaned from CPB on dopamine (initially at 5 mcg/kg/min, range 0–5 mcg/kg/min) and milrinone (0.5 mcg/kg/min). Nitroprusside 0.5 mcg/kg/min, titrated to effect, was used briefly for a period of hypertension. Dexmedetomidine (0.7 mcg/kg/hr) was started during rewarming for post-op sedation. After successfully separating from cardiopulmonary bypass, O_2_ saturations were near 100% and the transpulmonary gradient (CVP-LAP) was 8 to 9 mmHg. The left subclavian venous line tip was visible in the superior vena cava, near the innominate vein-superior vena cava (SVC) junction. Modified ultrafiltration was performed, protamine administered, and routine decannulation and sternotomy closure were performed. The cardiopulmonary bypass time was 36 minutes.

During the postbypass period the patient had stable vital signs with invasive blood pressures (IBPs) of ~90/40, heart rates (HRs) of *∼*150, oxygen saturations of 80–90% on 100% FiO_2_, central venous pressures (CVPs) of 12, and transpulmonary gradient (CVP-LAP) of 7–9 mmHg. Cerebral and somatic saturations were unchanged from baseline and the prebypass period.

 Preparations were being made to transport the patient to the ICU when a sudden drop in SpO_2_ to the 60's, a sudden rise in CVP to the mid 20's, ST segment elevation, and an acute change in the patient's color (head and face became profoundly plethoric) were noted. EtCO_2_ was also markedly decreased although there was no change in ventilation. Bradycardia soon followed as oxygen saturations continued to decline. Cardiopulmonary resuscitation (CPR) was immediately started with chest compressions and administration of resuscitative drugs. Preparations for reopening the chest and initiating cardiopulmonary bypass (CPB) were made while CPR continued. Profound hypoxemia persisted despite good quality CPR, confirmed with an arterial pulse with compressions and adequate ventilation.

**Figure 2:**
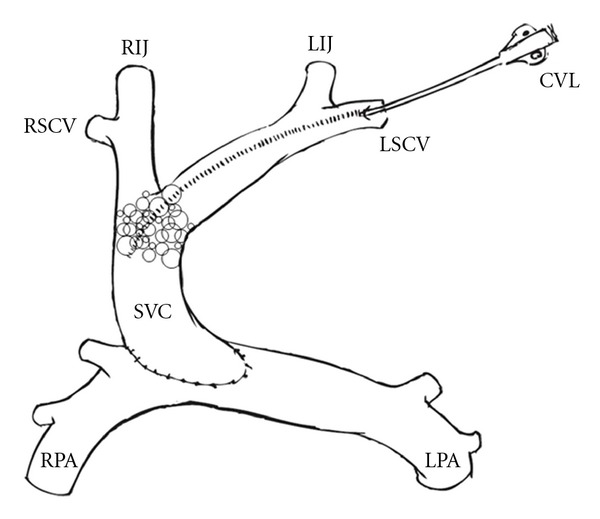
Depiction of surgical anastomosis with air bubbles at SVC-PA junction. (RSCV: right superior cava vein; LSCV: left superior cava vein; CVL: central venous line; RIJ: right internal jugular; LIJ: left internal jugular; SVC: superior vena cava; RPA: right pulmonary artery; LPA: left pulmonary artery).

The chest was reopened during the administration of CPR with no evidence of tamponade. On examination of the superior vena cava, an abundant collection of air bubbles was visible through the wall of the vein, appearing to fill the entire superior vena cava from the pulmonary anastomosis to the innominate vein (see Figure 2). A needle was placed in the superior vena cava and a large amount of air escaped through the needle hole. Myocardial function had begun to decline despite some improvement in oxygenation, so the patient was placed emergently on cardiopulmonary bypass. Sixteen minutes had elapsed since hemodynamic collapse and initiation of full CPB. No air bubbles in the systemic circulation were observed at aortic cannulation, offering some assurance that the emboli were confined to the cavopulmonary circuit. Approximately 3 mL of air was aspirated from the SVC while the patient was on CPB. The patient was separated from cardiopulmonary bypass after 36 minutes, with hemodynamics nearly identical to those before arrest, the exception being slightly lower oxygen saturations, 80–85%. During the bypass period, efforts were made to determine the source of the emboli and prevent further expansion, but no definitive source was ever discovered. Unfortunately, echocardiography was not used during the initial procedure, so it was not available at the time of the arrest.

The patient remained stable after the second bypass period and tolerated chest closure. Systemic hypothermia was initiated Intra-op and continued for 24 hours post-op with a target temperature of 34-35°C. He was taken to the PICU in stable condition. The patient did well over the remainder of his hospital course with no discernible neurological or cardiovascular sequelae of the arrest and was discharged to home on POD 10.

## 3. Discussion

Gas emboli are relatively uncommon, but not rare occurrences in the setting of pediatric cardiac surgery and usually occur during central venous cannulation, during sternotomy, while opening the heart chambers during cardiopulmonary bypass, or due to disconnects in the extracorporeal circuit. This patient's complex anatomy and the timing of events, with the arrest occurring after surgical closure, make this case unusual. Patients with cavopulmonary connections have passive pulmonary circulation and are dependent on venous return from the superior vena cava for all pulmonary blood flow. In this patient, the venous air embolism caused complete obstruction to pulmonary blood flow resulting in cardiopulmonary arrest. In patients with cavopulmonary anastomosis, venous return from the upper extremities and the head drain via the SVC to the pulmonary circuit, while blood return from the lower extremity and viscera drain via the IVC to the heart. This separation of venous return from the upper and lower body allowed us to narrow the source of air embolism to the upper compartment. Possible sources include the left subclavian central line or upper extremity intravenous line, accumulation of air from multiple medication injections throughout the case, residual air in the SVC from the anastomosis, or air trapped in the pulmonary arterial tree during the CPB period. Due to the acuteness of the arrest, we believe that it was a sudden bolus or entrainment of air from an intravascular line rather than an accumulation or residual air, although we are not aware of such a bolus of air occurring. We meticulously inspected all intravenous tubing following the arrest for loose connections, cracks, or disconnects that might have allowed air to be entrained but found none. We think residual air from the surgical procedure or air that might have been introduced during CPB, would have long since passed and is an unlikely source.

The event occurred as the child was being prepared for transport from the OR to the ICU, which is a time when the anesthesia provider is distracted by the act of transferring multiple lines, monitors, and critical medical equipment. During this period of time, vigilance may be diminished, and the anesthesia provider may be distracted by the multitasking required to prepare the patient for transport. Yet, this remains one of the more critical times during the case, as the patient's anesthetic requirements and hemodynamic parameters may be fluctuating requiring titration of anesthetic levels and hemodynamic infusions. The anesthesia providers caring for this patient were experienced in caring for children with congenital heart disease and are cognizant of the need to de-air tubing and the need to aspirate for air prior to injecting, but it is possible that due to distraction or diminished vigilance air may have been injected or infused unnoticed.

A potential source that was explored was the pressure bag attached to the transducer for the CVP line. It has been our practice to leave the drip chamber half full to allow visualization of fluid dripping when the transducer is flushed. We also use the CVP transducer port as a medication push port and flush medications by using the pig tail on the transducer. If the pressure bag is inverted, as might occur as a patient is being transported, this can allow the air in the drip chamber to be flushed into the patient.

In summary, gas emboli are a rare but important complication of pediatric cardiac surgery, as they may have devastating consequences. This is an unusual case of gas embolism involving a patient with single ventricle physiology after cavopulmonary connection leading to complete obstruction to pulmonary blood flow and cardiopulmonary arrest. Venous air embolism has not previously been reported as a cause of hypoxia following cavopulmonary connection. The exact etiology and source of the emboli in our patient remains unknown although presumably it was introduced into the circulation via an intravascular catheter. Prevention of gas emboli requires vigilance on the part of the entire perioperative team. Successful outcomes depend on prompt recognition and aggressive treatment, with the goal of treatment being to reduce the size of the embolus and minimize end-organ damage. In this case, resuscitation and outcome was successful due to the immediate recognition, good quality CPR and immediate access to hypothermic CPB.
